# Understanding multinational companies in public health systems, using a competitive advantage framework

**DOI:** 10.1186/1744-8603-7-19

**Published:** 2011-07-01

**Authors:** Jane Lethbridge

**Affiliations:** 1 Principal Lecturer, Public Services International Research Unit (PSIRU), The Business School, University of Greenwich, UK

## Abstract

**Background:**

This paper discusses the findings of a study which developed five case studies of five multinational health care companies involved in public health care systems. Strategies were analysed in terms of attitude to marketing, pricing and regulation. The company strategies have been subjected to an analysis using Porter's Five Forces, a business strategy framework, which is unusual in health policy studies.

**Methods:**

This paper shows how analysing company strategy using a business tool can contribute to understanding the strategies of global capital in national health systems. It shows how social science methodologies can draw from business methods to explain company strategies.

**Results:**

The five companies considered in this paper demonstrate that their strategies have many dimensions, which fit into Porter's Five Forces of comparative advantage. More importantly the Five Forces can be used to identify factors that influence company entry into public health care systems.

**Conclusions:**

The process of examining the strategic objectives of five health care companies shows that a business tool can help to explain the actions and motives of health care companies towards public health care systems, and so contribute to a better understanding of the strategies of global capital in national health systems. Health service commissioners need to understand this dynamic process, which will evolve as the nature of public health care systems change.

## Background

Multinational company involvement in public health care systems has been evolving since the late 1980s/1990s, with the introduction of compulsory competitive tendering for services such as catering, cleaning and facilities management services. For some companies, this formed the springboard for involvement in formal public-private partnerships for capital projects [1]. However in these two phases, the multinational companies were more likely to be service, property and finance companies, rather than health care companies. More recently, healthcare multinationals have started to become involved in public health care systems as providers of health care [2]. This paper explore the processes involved in this development, which can be argued in another variant of public-private partnerships-or even a further stage in a typology from marketisation to privatisation [3].

This paper aims to explore how a group of health care multinational companies have become part of several national health care systems over the last decade. The characteristics of this group of new global players are varied and reflect the national origins of many companies. They include experience of delivering acute, mental health services, and care services for older people to public providers at national levels, vertical integration of renal care services, and high technology care. Much of the expansion has taken place in Europe, during the last decade. The expansion of renal care companies and high technology care is a more global expansion. Expansion into older care services is beginning to have a global impact in countries with an ageing population.

Understanding this process of integration into public health systems will help to provide insights into the strategies that global capital is using to access national health systems. This paper will use a competitive advantage framework to gain an understanding of how these companies have moved into the public sector, which will contribute to the development of new health policy methodologies to study public-private relationships.

## Methodology

The research question underlying this paper is "How and why can global private interests enter national health policy settings? The hypothesis that emerges from this research question is "That global private interests enter national health policy settings through the use of expertise and additional skills for national health systems dealing with growing demographic pressures and demands for health care."

How to research the involvement of multinational health care companies in public sector health care systems raises a number of methodological issues which need to be considered in wider debates about globalisation and health. Much qualitative social science research has focused on the processes of understanding research subjects and so creating an understanding of different forms of social reality. Social science research, throughout the twentieth century, has gradually evolved ways of exploring different social realities of communities connected to health care, whether patients, health professionals or health institutions as forms of organisational culture.

Exploring the actions of multinational companies in relation to their growing role in public health care systems raises questions about whether to consider company activities in a business or a sociological context. Do we want to explore the 'life-worlds' of multinational companies? Initial research that explored the strategies of five multinational health care companies used ten key informant interviews, supported by analysis of company publications and evidence from market research reports, published academic research on aspects of contracting, regulation and pricing at a global and national level, and the press [1].

Business research tends to ask questions about how and why companies take certain decisions and the implications of these decisions for company competitiveness and profits. As a form of applied research, business research can be used by companies to inform future strategies. This paper argues that research into how multinational health care companies are becoming integral parts of public sector health systems, has to engage with some of the strategic methodologies that companies use to analyse their competitive environments. This can be seen as a form of interpretivist research in that it tries to understand decisions from the perspective of this new group of stakeholders, within the public sector. Rather as research into patient experience starts by trying to understand the 'world' of the patient, this research aims to start to understand the world of the multinational health care company, through a competitive advantage framework.

As the number of health care multinational companies involved in public healthcare systems is growing slowly, it was decided to use a case study approach, with five companies chosen to illustrate different scenarios. These companies have been chosen because they fulfilled one or more of these five basic criteria:

• Extensive experience of working with public health systems

• Moving into health care from service sector

• Moving from one region into global markets

• Expanding in one region with strong public sector

• Expanding in one region with weaker public sector

1. A Swedish company, Capio, was chosen because it was starting to expand outside the Nordic market that it was currently active in. The Nordic region has a strong public sector focus for health service delivery. Studying how a company expanded in countries with a strong public sector would provide insights into how a company articulated its strategy for working with a public sector.

2. ISS Healthcare, was chosen because the large parent company, ISS, was already involved in global facilities management business in the public and private sectors. This multinational company had set up a health care division as a way of entering a more specific health care market and would provide evidence of how a services company moved towards healthcare contracts.

3. A German health care MNC, Fresenius, was chosen because it was expanding globally and because it has a vertical range of health care products from kidney dialysis to health care delivery. It might illustrate different strategies for entering public health care markets.

4. An Asian company, Parkway Holdings, was chosen because it was expanding in a regional market in Asia, where public health systems have different remits and scope.

5. A well-established, UK based company, BUPA, was chosen because of its move from UK market to international market, particularly Asia.

A qualitative approach was used to assess strategic developments. Data was gathered through both interviews and document analysis. A question guide was drawn up which covered topics such as approaches to overall strategic development, marketing strategies, perceptions of the external environment, relationships with public health systems, and attitudes to contracting, pricing and regulation. As an additional stage of data gathering, the company strategies were tested after five years to see if they had been met and to what extent these strategies has changed over this period. This was done through an analysis of company documents during the five year period.

Face to face interviews were conducted by Jane Lethbridge in Europe and Loh Foon Fong in South East Asia. One interview was conducted by telephone. Other data was gathered by Jane Lethbridge. Data analysis was conducted by Jane Lethbridge. Potential interviewees were approached by e-mail, with an outline of the UNRISD project and a copy of the question guide. A list of respondents is attached as an appendix. Interviews were recorded by tape recorder, with additional notes taken during the interviews to highlight what were felt to be important issues and which acted as prompts during the interview. The interviews were transcribed.

Grounded theory informed the interview analysis. The text of the interviews was coded which helped to identify the major concerns of respondents. These were compared to the key themes that had been used to inform the development of the question guide. These two groups were then compared and the codes were re-grouped to form concepts. The document analysis was approached by looking at company and related reports, over a 10 year time period. The company reports were subject to an analysis of key developments that had taken place during this period, particularly indicators of expansion or contraction within a market. The results of newspaper searches of the period were used to explore mergers and acquisitions.

Models of business strategy have evolved over the last few decades. The essential core of business strategy is to try to assess and plan for competitive advantage. There are three main approaches to strategic management. The oldest approach uses an input/output model to assess outcomes in a competitive external environment. A second approach looks at a resource based view of the company. A third and more recent approach is knowledge management [nine]. This paper will focus on the assessment of the competitive external environment because this will help to explain how companies are viewing public sector health systems. It is the interpretation of a public health care sector environment through a competitive lens, which will contribute to a greater understanding of company strategies.

**Figure 1:**
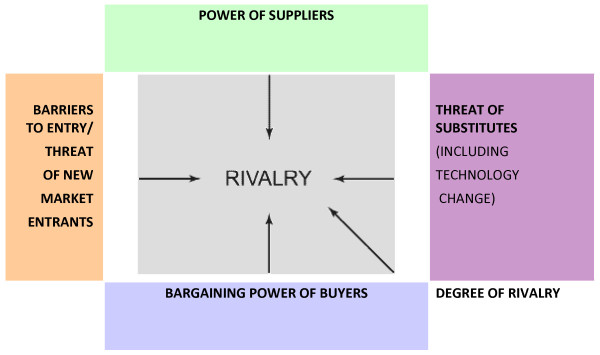
**Porter's Five Forces**. (Porter, 1980).

One of the foundations of modern business strategy, which provides a model to assess the external business environment is Porter's Five Forces theory of company expansion [4,5]. Porter's Five Forces of company expansion will be used as a framework for the strategic analysis of the five multinational health care companies, which were the subject of research five years ago. Porter looks at the interdependence of dynamic factors in company expansion, particularly competitive advantage. His theory of company expansion has five basic elements (Figure 1).

This paper is testing the use of Porter's five forces model as a way of achieving a better understanding of multinational health care company strategies. In the context of exploring different social science methodologies for the study of health policy and health systems, Porter's Five Forces model has had a great influence on strategic management. Porter is considered to have provided strategic management with a theoretical framework, which is accessible to both academics and practitioners [6]. In this sense, it was considered an appropriate model to analyse multinational company behaviour in the health sector.

There have been some isolated attempts to apply Porter's model on competitive advantage to the health care industry. Pines (2006) applied the model to emergency medicine in the United States [7]. Emergency medicine is unusual in that it has to provide continual access to the health care system. The analysis showed the precarious position of emergency care but highlighted how emergency medicine physicians could campaign for change to strengthen their position. Sheppard (1997) applies Porter's Five Forces to the Australian physiotherapy industry [8]. This analysis concluded that physiotherapy should reposition so that clients were viewed as both suppliers and recipients of care, in order to gain competitive advantage.

Breedveld et al (2006) used an adapted model of Porter's Five Forces to examine the Dutch home care industry [9]. The study concluded that two additional factors, the role of government and relations with suppliers of complementary products or services, should be included in order to explain how the home care industry operated. This relatively small group of studies, where Porter's Five Forces have been applied in a health care context, are primarily concerned with different national health care settings, such as emergency medicine. The application to public health care service services is limited. Gaining insights into how private providers, particularly international health care companies, view the potential of national health care markets, will contribute to a greater understanding of how changes in public sector health care policy affects company behaviour.

In this study, Porter's Five Forces have been used to identify key factors that have real or potential impact on company entry into national health systems, by understanding approaches to strategic comparative advantage. These factors can be used by public health systems to impact of companies. Porter's Five Forces have been translated into key factors, which are set out below.

• Existing competitive rivalry-The importance of ownership

• Barriers to entry-Gaining influence

• Bargaining power of buyers-Exploiting national differences

• Power of suppliers-Making adjustments

• Threat of substitute products-Providing expert capacity

This provides an indication of how further strategic public-private partnerships are being constructed by the private sector. The findings are set out in the following section.

## Findings

Existing competitive rivalry-The importance of ownership

Market growth for five health care multinational companies perceived market growth in two ways, and both were dependent on finding ways for patients/consumers to pay for health care. In the conventional private sector health care market, health insurance is a key element necessary for private health care expansion unless depending on private direct payers. A much more significant finding in the 2005 research was that health care companies viewed the public health care sector as an expanding market, albeit a new and different one. The rate of outsourcing of services from the public sector, including clinical services, has increased and thus provides an expanding market. Contracting out of clinical services and high technology diagnostic testing has increased as a result of legislation and health policies that increased the role of private providers in providing 'choice' for patients. Company strategies reflected this analysis. Capio wanted to "*become a pan-European operator, playing a role in the restructuring of the health care sector; *to focus on *"the provision of acute health care sector": *and to *"develop further focused service lines e.g. ophthalmology, oncology, cardiac surgery, orthopaedics*. ISS healthcare aimed to expand into the Nordic market with specific medical specialties.

Rivalry in health care industry manifests itself in many ways, particularly in the context of the public health care sector. Perhaps the issue of price is the most important. Health care companies have found that the lack of transparency of public health care prices made competing more difficult. In 2005, the introduction of diagnostic or health resource group (DRG/HRG) systems of pricing, by the public sector, was considered a positive development by the five health care companies. Diagnostic related groups (DRGs) are a system of categorising patients based on diagnosis, treatment/procedures, age and length of stay. Categories establish a uniform cost of each category and a maximum price for reimbursement. Medicare, the United States government health insurance programme, originally introduced DRGs in 1983 as a way of trying to control the Medicare budget [10]. These new national systems of diagnostic/health related groups have been more widely adopted over the past five years in relation to resource allocation and pricing. It also enables international comparisons to be made [11]. In the United Kingdom, pricing by health related groups or 'Payment By Results' has been implemented in acute services, since 2005 [12]. In Nordic countries, diagnostic related groups have become the basis for pricing and are considered to improve quality through higher prices or extra payments [11]. In Germany, the new system is considered to increase competition between hospitals [13].]. One of the main concerns felt by four companies was that they would only be able to compete as a result of transparency in pricing. Their criticisms of the existing DRGs systems, in Europe, were based on a view that governments were setting pricing systems that were still biased towards public providers.

Changes in ownership are an indication of how rivalry is operating in the health care industry. In the last five years there have been some significant changes in ownership experienced by the five companies. The most significant changes in ownership were experienced by Capio and ISS, both Nordic companies. In September 2006, Capio was bought by Opica, a company "*indirectly jointly owned by Apax Partners Worldwide LLP, by Nordic Capital Fund VI and by funds advised or managed by Apax Partners SA*". The company was de-listed in November 2006. Opica AB is jointly owned by Apax Europe VI (45%), Nordic Capital Fund VI (44%) and Apax France (11%). This acquisition was conditional on Capio selling its UK hospitals in order "*to avoid regulatory problems*" [14]. In June 2007, Opica was given permission by the European Commission to sell Capio UK [15]. Capio UK was sold to Ramsay Health care, an Australian health care company, in 2008. This shows that the European Commission (EC) was a player in this process. Between 2007 and 2010, Capio also sold its hospitals in Finland, Denmark and Switzerland, but achieved its goals of moving into Spain and Germany, as well as consolidating its presence in France.

In February 2005, ISS sold its health care operations and its 49% interest in CarePartner to a joint venture, now named Aleris Holding AB, owned by ISS EQT III ltd and Aleris's management. ISS made a profit of DKK123 million from this sale. At the end of June 2005, ISS sold its interest in this joint venture and made a further profit of DKK 114 million. In 2005, ISS itself was bought by PurusCo A/S, a consortium of EQT (a Swedish private equity company) and Goldman Sachs Capital Partners and was de-listed from the Copenhagen Stock exchange. As with the Capio sale, the European Commission was involved as a competition regulator in this process.

The case of Parkway ownership is a much more fluid situation. In 2005, Texas Pacific Capital (TPG) bought 26% of Parkway shares [16]. In March 2010, TPG sold 23.9% of shares to Fortis International, an Indian health care company, interested in international expansion. Within weeks, Khazanah Nasional Berhad, the Malaysian government investment arm, and existing Parkway shareholder, had made a bid for ownership. In June 2010, these two shareholders were struggling to control Parkway but by July 2010, Khazanah Nasional Berhad was successful in gaining control of Parkway [17]). This sale was not subject to the same regulatory competition rules that exist in Europe. The overall ownership of both BUPA and Fresenius remains the same in 2010 as it was in 2005 but they have both been involved in acquisitions to enter new markets and implemented some rationalisations. Although BUPA remains a non-profit company, it sold 25 acute hospitals to Cinven, a private equity investor, for £1.44 billion in 2007, in order to pay off debt and to focus on long term development of the company, internationally and in the care sector [18]. As a company in a country that is part of the European Union, it will have been scrutinised by the European Commission Since 2007, the company has expanded it health insurance and care activities globally. It bought the Amity Group in Australia and Guardian Health care in New Zealand. It also entered the US market in 2008 with the acquisition of Health Dialog, a company providing health care analytics and decision support services to 19 million people in the US and UK, Spain and France. However, in 2009, BUPA acquired the Brompton Hospital, which is based in London and serves the international market, rather than the UK market [18].

As part of its strategy to expand into health care management, Fresenius bought the HELIOS group, a German private hospital group, which has 55 hospitals and 26,000 employees in Germany. This became an internal Fresenius division. The Wittgensteiner Klinken Group, which Fresenius bought in 2001, has been integrated into the HELIOS division. There are further signs of internal change within Fresenius with the acquisition by Fresenius Vamed, the international health management division, of four clinics in the Czech Republic, from Fresenius Helios. Fresenius Medical Care, the dialysis clinics division, bought the Renal Care Group, a company providing kidney dialysis, in the United States in 2006, thus expanding its presence in North America [19].

Although the four companies have different patterns of sales and acquisitions, they show that there is a constant process of reviewing what is profitable and what is considered important to test out. Both Capio and BUPA have sold a large part of their portfolio in order to pursue their strategic objectives. Fresenius is also involved in internal re-organisation, moving assets between divisions, which it reviews systematically. This suggests that ownership is one of the most important survival strategies for global players. The European Union competition policy has influenced some of the changes in ownership, often calling for the sale of national division of a company to ensure competition is maintained between member states. The extent to which national governments have been involved in these sales and acquisitions is unclear. However some of these examples show that a public health system may contract a company to run services but have little influence over potential changes in ownership of that contracted company.

Historically, health competences at EU level have been developed to promote a common market. Other aspects of health policy have evolved as a result of policy developments in related fields. Health policy has traditionally been caught between the EU Treaties implemented through European legislation and the European Court of Justice (ECJ), and policy making, which has been consensual between member states. Recently, the ECJ has had an influence on health policy in the fields of health care, medicines, environment, workplace health and safety and pharmaceuticals/distribution. Health care has been most strongly influenced by the concept of subsidiarity with national governments considering national health care systems to be their own responsibility [20]. Increasingly EU pressures, as well as influencing public health crises, are influencing market integration and compliance within national health care systems. The European health policy process is essentially issue-specific, fragmented and incremental [21]. Greer argues that health care systems face potential problems with the extensive network of the competition and other related legistation [22]. The influence of competition policy on company takeovers is one example of how health systems can experience the impact of the EU internal market.

### Barriers to entry-Gaining influence

The barriers to entry into an industry are the unique characteristics of an industry. In the case of the public health care sector, the barriers are most often created by government and public policy. In 2005, several multinational health care companies acknowledged that understanding the public sector was a major barrier that restricted their entry. In this sense, the development of a knowledge base about how to operate within the public sector environment is key to entering the market.

The development of new ideas and knowledge that address some of the problems facing public health care is also necessary and can be used as a way of understanding the market. The engagement of companies in new ways of delivering care, offering expertise and providing case studies, which will help the public sector to improve efficiency, is also a contribution to addressing the perceived problems of an ageing society. This can be interpreted as part of company strategy to enter a new market and to manage political risk. Fresenius saw 'innovation, market leadership and products and services' as necessary for becoming a large international health care company. Innovation was considered the 'nucleus for growth'.

BUPA illustrates a different strategy for entering a new market. It has created several partnerships to enter new markets and specific parts of national markets. In Spain, BUPA has built on its partnership with SANITAS to enter the public health care market, leading to a public-private partnership hospitals initiative. In the United Kingdom, its strategy to enter the NHS was part of long term strategy, which started over a decade ago, which has not always been successful, because the UK government was focused on encouraging international companies to become providers, rather than existing UK private companies [23]. Its acquisition of Health Dialog, a United States company involved in health information systems, will place the company in a good position to take over commissioning functions in the NHS, recently announced in July 2010.

As hospital assets are difficult to use for anything other than health care delivery, companies have shown signs of being cautious about investing in buildings and other capital developments unless they are in partnership with other companies. As a way of understanding the diversity of rivals and other health care cultures, Fresenius VAMED, its international health care division, is developing extensive experience of understanding other health care systems. This is developing through project development, consulting, planning, project management and construction. Over 75% of its sales in these services are in Europe [24]. Many of these services are being sold to the public sector. This will improve the company understanding of public sector cultures, as a way into future markets. Public-private partnerships can also be considered as a way of controlling the power of buyers and will be discussed in the next section.

### Bargaining power of buyers-Exploiting national differences

Buyers in the public health care sector are primarily public sector commissioners rather than direct consumers of health care. Private companies need to understand how the bargaining power of commissioners operates and identify the knowledge that is needed to operate effectively in this environment. In the case of public-private partnerships, the concept was new to both the public and private sectors when they were introduced almost twenty years ago. Three companies, BUPA, CAPIO and Fresenius, show that public--private partnerships, on a long term basis, have become an important part of company strategy. They illustrate how national government policies influence company decisions. The impact of these changes on national health care systems varies according to the historical development of public health care systems and the role that the private sector has played in health care provision.

Capio was one of the first companies to take over the management of a public sector hospital in Sweden. Since then, Capio has negotiated longer contracts at St. Gorens, Stockholm, Sweden and Valdemoro, Spain. The contract for Valdemoro, Madrid, is a contract on a capitation basis, which provides an annual fixed payment per head of population. The contract involved building a hospital, which opened at the end of 2007 [25]. The contract is for 30 years.

BUPA's major European subsidiary is Sanitas, a Spanish health insurer and health care provider, which was incorporated into BUPA in 1989. In September 2006, Sanitas, won a government tender to build and run a large new public hospital in Manises, Valencia. The 15 year private finance initiative contract, for the Valencia government, involves building and managing the new hospital as well as updating and running primary care centres in the region. Sanitas has formed a consortium with Ribera Salud, which is owned by Bancaja and Caja Mediterraneo, two Spanish banks [26]. The project is worth €137 million [27]. This initiative is significant for BUPA in that it is in Spain rather than the UK. The Spanish national health system has been created in the last twenty years and has adopted many strategies of health sector reform in this process. It is a newer system, which has adopted policies with fewer constraints from the existing health care system. In the Spanish context, BUPA is perceived as an international company, which is already involved in health insurance.

Fresenius, through VAMED, its international health care division, uses public-private partnerships to expand it health care management expertise internationally. It has become involved in public-private partnerships in Austria and Bosnia. In Bosnia, VAMED is modernising the University Hospital at Tuzla and building a new medical centre at Banja Luka. VAMED is also contracted to deliver technical management for the Vienna General Hospital and manages the non-medical services contact for the Charite University Hospital, Berlin. Fresenius has organised its international health care division as separate from German hospital management division, a reflection of their different strategic roles in company expansion. The German government has adopted a more direct policy of heath care privatisation by selling several public hospitals to private companies. Fresenius identified opportunities that were emerging from "*the expected privatisation of health systems" *and would be able *"to build up a company that can command a leading position in the hospital field"*.

Health care Europa, a subscription newsletter, which has been set up in partnership with Laing & Buisson, to provide information on the private health care industry in Europe for investors, provides an interesting perspective on public-private partnerships and hospitals concessions. In a recent article on the Alzira public-private partnership, one of the first public-private partnerships in Spain, it reports that the returns are 1-2%. Health care Europa reports that with returns at this low level, the private companies are gaining experience of '*how to run health care really efficiently. In that sense, the concessions are private health care labs for Europe as most of these insurers are international in scope and active in many countries*' [28]. From an investor perspective, public-private partnerships are not necessarily high yielding products but are often maintained over a long time scale.

The question of whether buyers or commissioners can switch easily is pertinent to health care. The length of contracts varies from one to thirty years or more. From the commissioner perspective, the understanding of how contracts should be managed and monitored has led to an increased awareness of the inflexibility of contracts. For a private health care company, the slow rate of switching is a perceived advantage, although this depends on the type of service. Public-private partnerships for hospital construction are examples of where switching is difficult. High technology contracts are more flexible, with recent examples of companies having contracts terminated because of poor quality services.

### Power of suppliers-Making adjustments

The definition of suppliers in the health care industry can include pharmaceutical industries, diagnostic and laboratory testing as well as labour supplies. The case of Capio shows how a health care company re-thought the supply of diagnostic testing during this period, which has some relevance to the public sector. Originally Capio had its own diagnostics division. After merging Capio Diagnostics with Capio UK in 2007, Capio Diagnostics took over UNILABS, a Swiss based company laboratory testing company in 2008. After rebranding as UNILABS, it was sold by Capio and now operates in eleven European countries. The UNILABS Board of Management has directors from both Apax Partners and Nordic Capital, which both own Capio [29]. Diagnostic testing is a discrete service which in some countries has already been contracted out from the public sector, but in other countries, has not yet been subject to a competition process. In this context, how companies approach the issue of suppliers is influenced by the national context in which they are operated. However, pressure on costs is making this service subject to cost reductions [28]. The sale of Capio's diagnostic services suggests that although it was a sale, it was also part of a process of changing policy towards one type of supplier.

One of the most important suppliers for health care companies is labour. Ensuring a supply of labour is a crucial factor for a labour intensive industry. In this context, controlling the training and education of health workers, by setting up training schools, is an example of how health care companies are trying to influence the supply of labour. Both Parkway and Fresenius were planning to establish training schools for nurses and other health care workers in 2005 and had both achieved this goal by 2010. Their Schools of Nursing supply their companies with trained staff [24,16]. In 2005, all companies viewed involvement in training and education, as an important way of gaining status and recognition within public health care systems.

Another approach to ensuring a supply of labour is to create favourable terms and conditions for employees. Two companies show that improving terms and conditions is part of their strategy. Capio completed negotiations for a European Works Council (EWC) in 2006. The European EWC Directive (1994) requires transnational companies to establish information and consultation agreements covering their entire European workforce [30]. The content of these agreements is largely left to negotiation between management and employee representatives, but minimum requirements where management refuses to negotiate, include the requirement of annual reports to the EWC on the company's business prospects, and the right to be informed about exceptional circumstances affecting employees' interests, such as closure or collective redundancy. The EWC directive applies to companies, or groups of companies, with at least 1000 employees across the member states, *and *at least 150 employees in each of two or more distinct member states. Fresenius has also established an EWC but more recently, Helios, its German hospital division, has negotiated its first trade union wage tariff agreement with ver-di, the services trade union, in 2006.

The group wage tariff with ver-di was expanded to cover non-medical staff. Eventually all Helios clinic staff will be integrated into the group wage tariff agreement [24]. This is an example of how a company tries to ensure a supply of labour in one country, for an activity which is labour intensive.

These examples of how companies deal with the power of suppliers are not restrictive to the public health care sector. They should be seen as part of a wider process of dealing with suppliers to gain competitive advantage.

### Threat of substitute products-Providing expert capacity

The threat of substitute products in the health care context raises questions about the future of health care and the possibility of different ways of delivering care. In relation to the public sector, multinational health care companies are considering substitute products in the context of new ways of delivering health care that are effective and cost-efficient. The re-programming of acute services so that they are delivered either through out-patient services or home care services is perhaps the most significant threat of a substitute health care product in the public sector. This is identified as the most important factor that is resulting in multinational health care presence in public health systems. For European national governments, how to deal with the growing demand for health care from an ageing population is a major challenge. Companies that can provide new and cheaper ways of delivering health care will be listened to with interest. This factor will have most influence on future public-private partnerships in health care.

BUPA has been working, in the United Kingdom, with the NHS on a 'Collaborative Care Model', which consists of an analytical tool, showing clinical risk profiles of populations, and health coaching. The Model aims to help healthcare providers target provision and improve the value of that provision by helping patients make the most of the care they receive and be more active in managing their chronic conditions. This initiative draws on expertise developed by Health Dialog in the United States [31]. Through this 'Collaborative Care Model', BUPA is developing two types of substitute products: one for commissioning, drawing from American experiences, and one for patients, encouraging them to self-manage their conditions.

This contributes to making commissioning more efficient but has the potential to change the way in which care is delivered to the patient, if the patients is playing a greater role in self-treatment. Neither of these approaches is unique but BUPA has packaged them together in this new initiative. It places the company in a position of offering new forms of support or substitute products to the NHS. It is involved in piloting and evaluating the health coaching initiative and has also developed resources to train and support nurses in their 'health coaching' role.

Capio has played a similar role in Sweden in the development of more effective ways of delivering acute care outside of the hospital setting. Aleris, which now operates in the Nordic region and delivers a range of primary, social and community care, can be seen as playing a role in the development of substitute products. With the focus on personalised care, the ability of a company to develop specific packages of care will influence its competitive position [32]. Aleris has been active, together with other private providers that deliver care to the public sector, in developing new services such as patient hotels, home care and new forms of nursing care. These are now being purchased by the public sector in the four Nordic countries.

These examples show that companies are aware of the need to develop substitute products that they can provide for public health care systems. The current political climate in Europe, where there is extensive questioning of how health care can be delivered to a growing older population, provides a good environment for companies to develop new forms of health care delivery. In addition, the development of substitutes should also be seen as part of a strategy for managing the political environment. Greater company involvement in rethinking the delivery of care will lead to raising their profiles as innovators, known to the public health care sector. Capio reflect this attitude to playing a role in improving health systems. In 2010, the company wants to "renew European health care and strengthen the individual's position by uniting quality, patient safety and efficiency. The ambition is achieved by offering cost-efficient medical services and standardised care processes with high quality." Fresenius would like to "become a leading global provider of products and therapies for critically and chronically ill people". This will place them in a stronger position to win contracts for new types of substitute services.

## Conclusion

Porter's Five Forces provides a useful framework to analyse company strategies but a practical benefit is that it can be used to identify factors that public health systems need to understand if they are to become effective commissioners. What has emerged from this analysis is that companies have identified the provision of expert capacity as one of the most important company strategies for their future partnerships with the public sector. Public health systems need an input of innovative ideas to address ways of responding to an increased demand for health care. The language of company strategies is beginning to use terms such as people's health, processes of renewal and choice of partners for the public sector, which reflect their positioning in a public health context. As well as providing expert capacity, companies will also be concerned with ways of gaining influence, which may not necessarily be directly related to a profitable activity. Companies may exploit national differences in health care systems and national health care systems need to be aware of when developing longer term partnerships with the private sector. How companies deal with suppliers of essential inputs, especially labour, is another issue that health care systems need to anticipate because they will affect the ability of the company to deliver health care.

The five companies considered in this paper demonstrate that their strategies have many dimensions, which fit into Porter's Five Forces. Several companies have undergone significant changes in ownership in the last five years, which did not lead to any significant changes in their overall strategies. There are some consistent goals in their strategies for 2010. The move that BUPA took by selling its acute hospitals was hinted at in its objective to focus on influencing people's health, rather than acute care. In its objectives for 2010, there is a similar objective to help people live healthier lives. Capio is still concerned with 'renewing European health care' with a focus on the individual. Aleris is still specific about wanting to be a partner with the public sector. There is a hint that Fresenius is focusing on its core business of renal care services but expanding to other critically ill groups, although there is no mention of health management. It remains an expanding global company. Parkway has not changed in its overall goals but its recent current ownership struggle may influence its future.

For multinational health care companies working with the public sector, the political management of risk is central to their positioning in the sector. However, in the current climate where the size of the public sector may shrink, the potential role of multinational companies may expand by taking on a greater number of contracted services or may decline because services will not be funded by the public sector at all. This could then lead to increase in use of supplementary health insurance and co-payments in public health care systems.

## Competing interests

The author declares that they have no competing interests.
